# Bee venom-derived antimicrobial peptide melectin has broad-spectrum potency, cell selectivity, and salt-resistant properties

**DOI:** 10.1038/s41598-020-66995-7

**Published:** 2020-06-23

**Authors:** Su Jin Ko, Eunji Park, Alina Asandei, Jee-Young Choi, Seung-Chul Lee, Chang Ho Seo, Tudor Luchian, Yoonkyung Park

**Affiliations:** 1 Department of Biomedical Science, Chosun University, Gwangju, 61452 South Korea; 2 Department of Physics, Alexandru I. Cuza University, Iasi, Romania; 3 Departments of Dermatology, Chonnam National University Medical School, Gwnagju, Korea; 4 Department of Bioinformatics, Kongju National University, Kongju, 32588 Republic of Korea; 5 Research Center for Proteineous Materials, Chosun University, Gwangju, 61452 South Korea; 6 Interdisciplinary Research Institute, Sciences Department, Alexandru I. Cuza University, Iasi, 700506 Romania; 7 Chonnam National University Hospital, Gwangju, Korea

**Keywords:** Antimicrobial resistance, Infection, Peptides

## Abstract

Antimicrobial peptides have attracted attention as alternatives to conventional antibiotics. Previously, a novel antimicrobial peptide, melectin, consisting of 18 amino acids was isolated from the venom of a bee, *Melecta albifrons*. Here, we investigated the antibacterial activity of melectin against drug-resistant bacteria. Melectin showed broad-spectrum antimicrobial activity but low cytotoxicity and no hemolytic activity. Melectin maintained its antimicrobial activity at physiological salt concentrations. Melectin is an α-helical structure that binds to the bacterial membrane via electrostatic interactions and kills bacteria in a short time by bacterial membrane targeting. Collectively, our results suggest that melectin has antibacterial activity and anti-inflammatory activity.

## Introduction

Excessive use of antibiotics leads to the development of drug-resistant bacteria, which threatens human health^1^. Drug-resistant bacteria develop rapidly, while novel antibiotics are discovered at a slower rate. To combat drug-resistant bacteria, new antibiotics must be developed. Antimicrobial peptides (AMPs), known as host defense peptides, consist of short amino acid sequences containing both positively charged and hydrophobic amino acids^2^. AMPs are attractive candidates as therapeutic agents, as they can kill a broad range of bacteria including antibiotic-resistant strains and do not tend to develop drug resistance^3^.

Most AMPs display antimicrobial activity by disrupting the bacterial membrane. The cationic charge of AMPs enables an electrostatic interaction with the negatively charged bacterial membrane. The cytoplasmic membrane of bacteria is rich in phospholipids phosphatidylglycerol, cardiolipin, and phosphatidylserine which have negatively charged head groups and bind to positively charged AMPs. Additionally, the presence of lipoteichoic acid (LTA) of gram-positive bacteria and lipopolysaccharide (LPS) of gram-negative bacteria acts as a lipophilic anchor^4,5^. AMPs can replace divalent cations such as Mg^2+^ and Ca^2+^ bound to LPS, causing membrane disruption and eventually bacterial death^6^. Some AMPs penetrate the bacterial membrane and kill bacteria without inducing bacterial membrane permeabilization. These AMPs attack DNA and RNA to inhibit protein synthesis^7^. Examples of these AMPs include buforin2^8^ and indolicidin^9^.

The salt sensitivity of AMPs is a major limitation to the development of AMPs as treatment agents^10^. AMPs interact electrostatically with the microbial membrane in a salt-sensitive manner. Human body fluid has a high salt concentration which interferes with the antimicrobial activity of AMPs^11^. Therefore, it is necessary to develop a peptide that maintains its antimicrobial activity even at physiological salt concentration.

Bee venoms contain diverse active compounds, including polypeptides, enzymes, and amino acids^12^. Bee venom therapy is the therapeutic application of bee venom. The compounds in bee venom are used as traditional medicines for anti-arthritis and pain relief^13^. Various antimicrobial peptides derived from bee venom have been reported, including mastoparan^14^, melittin^15^, apamin^16^, and secapin^17^, which have potent antimicrobial acitivites.

In a previous study, a novel antimicrobial peptide was isolated from bee venom and named as melectin. Melectin was the first peptide identified in the solitary bee venom. It is a cationic amphipathic peptide that contains rich hydrophobic and basic amino acid residues and a proline. Melectin has excellent antimicrobial activity^18^. Melectin can be useful as a new template for developing effective antibiotic peptides. In the present study, we evaluated the mechanism of action of melectin by conducting minimum inhibitory concentration (MIC) measurements against *Staphylococcus aureus*, *Pseudomonas aeruginosa*, *Salmonella typhimurium*, *Klebsiella pneumoniae*, *Escherichia coli*, and drug-resistant bacteria. The cytotoxicity effect was determined against fibroblasts, and the killing efficacy was measured in a time kill kinetic assay. We also evaluated whether melectin retains its antimicrobial activity in the presence of cations. To investigate the action of the peptide on the bacterial membrane, outer membrane permeabilization and cytoplasmic membrane depolarization were evaluated using a fluorescence reagent and flow cytometry.

## Results

### Characterization of the Peptide

**Figure 1:**
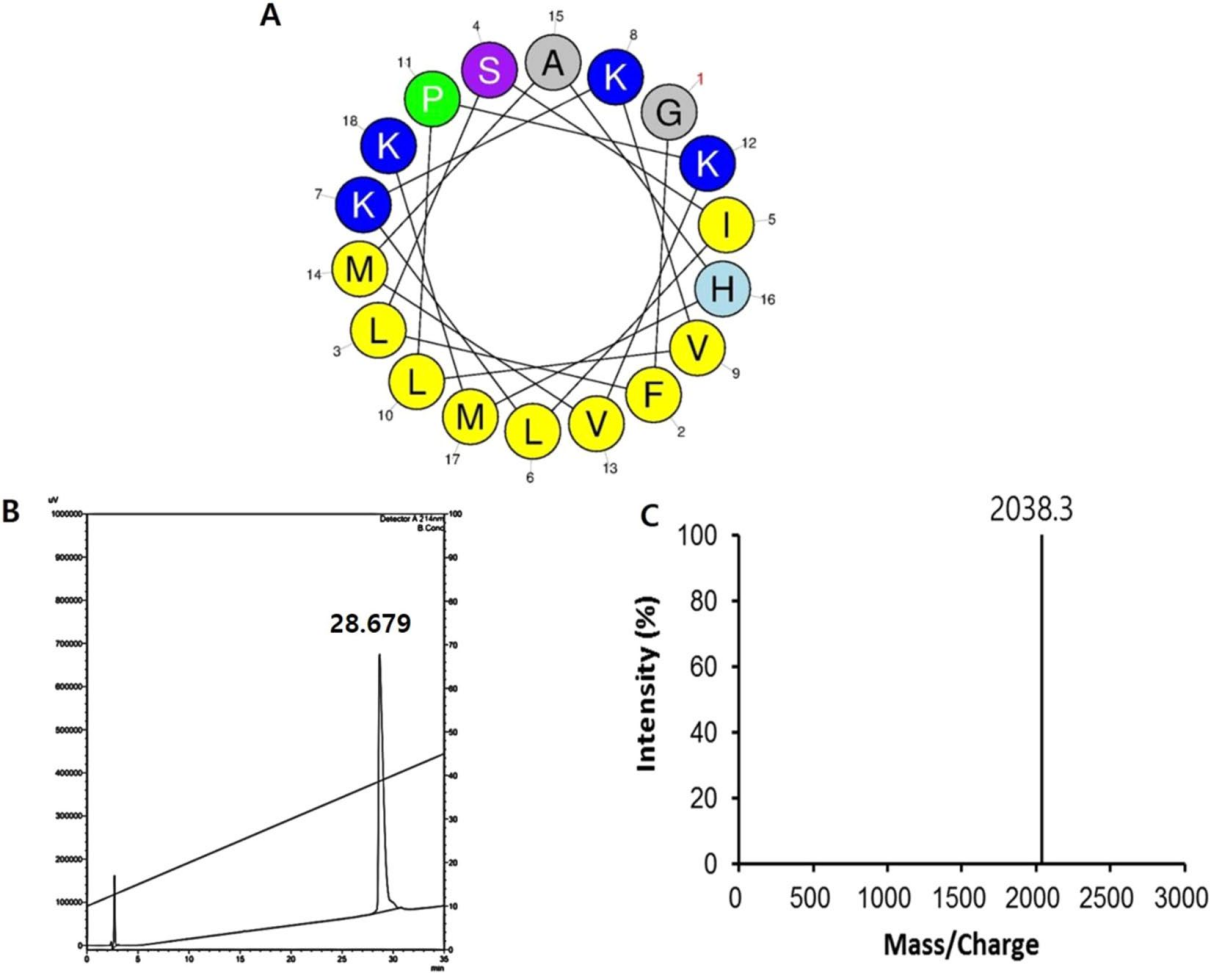
Helical wheel diagram of the peptide. (**A**) The projections were obtained from http://heliquest.ipmc.cnrs.fr/cgibn/ComputParam.py^41^. Yellow indicates hydrophobic residues. Blue indicates positively charged residues. Gray represents other residues. (**B**) Reverse-phase HPLC (RP-HPLC) profile on a C18 column with detection at 214 nm. The black arrow represents the retention time of melectin (29.960 min). (**C**) MALDI mass spectrometric analysis of the synthetic peptide. A major peak appeared at the m/z value of melectin (2038.3).

The melectin sequence, theoretically calculated and experimentally determined molecular weights, and retention time are summarized in Table 1. The observed molecular weight was consistent with the calculated molecular weight, indicating that the peptide was synthesized successfully. The identity of the synthetic peptide was confirmed by reverse-phase high performance liquid chromatography on a C18 column. The molecular weight of the peptide was verified by mass spectrometry (Fig. 1).

**Table 1:** Amino acid sequences and physicochemical properties of melectin.

Peptide	Sequence	Retention time	Net charge	Molecular weight (Da)	
				Calculated	Observed
Melectin	GFLSILKKVLPKVMAHMK-NH_2_	28.679	+5	2038.23	2038.3

### Antimicrobial activity

The minimum inhibitory concentration (MIC) is the lowest concentration that prevents bacterial growth. The MIC values of melectin were calculated as the concentration that inhibits more than 99% of bacterial growth and are summarized in Table 2. Melectin exhibited potent antibacterial activity against gram-positive and gram-negative bacteria strains including drug-resistant bacteria strains. Melectin showed antimicrobial activity of 2 μM against *S. aureus* and *P. aeruginosa* and 4 μM against *K. pneumonia* and *E. coli*. Antibiotics do not have antibacterial activity against the resistant strains (Table S1), while melectin had antibacterial activity at 2–8 μM.

**Table 2:** Minimum inhibitory concentration (MIC) of antimicrobial peptides against microorganisms.

Microorganisms	MIC (μM)	
	Melectin	Melittin
*Staphylococcus aureus* ATCC 25923	2	2
*Pseudomonas aeruginosa* KCTC 2004	2	2
*Klebsiella pneumoniae* KCTC 2008	4	2
*Escherichia coli* ATCC 25922	4	1
**Drug resistant bacteria**		
*S. aureus* MRSA 366	4	1
*S. aureus* MRSA 254	8	4
*S. aureus* MRSA 660	8	2
*P. aeruginosa* 3543	8	2
*P. aeruginosa* 1034	4	2
*P. aeruginosa* 5018	2	2
*E. coli* CCARM 1229	2	2
*E. coli* CCARM 1238	2	2

### Cytotoxicity and hemolytic activity

**Figure 2:**
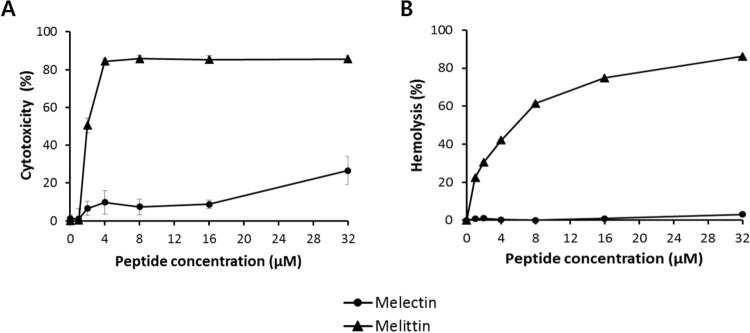
Cytotoxicity of antimicrobial peptides. (**A**) Human fibroblasts were used to evaluate the cytotoxicity of peptides. Cytotoxicity was measured through the MTT assay. (**B**) Hemolytic activity of the peptides against red blood cells. The graphs were derived from average values of three independent trials.

The cytotoxicity of melectin was evaluated using normal human fibroblast cells, with melittin used as a control. At 32 μM, melectin showed approximately 30% cytotoxicity and low cytotoxicity of 10% at concentrations below 16 μM (Fig. 2A). In contrast, melittin exhibited approximately 80% cytotoxicity at 4 μM. In the hemolysis assay, melectin showed no hemolytic activity at up to 32 μM, while melittin showed 80% hemolytic activity at 32 μM (Fig. 2B). As a result, melectin has low cytotoxicity and no hemolytic activity at its active concentration.

### Time kill kinetics

**Figure 3:**
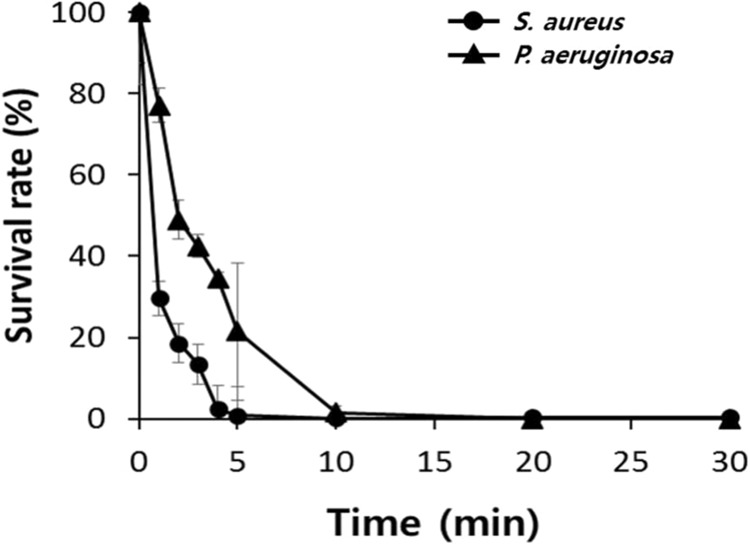
Time-kill kinetic curves of melectin against microorganisms. *S. aureus* ATCC 25923 and *P. aeruginosa* ATCC 27853 were exposed to melectin for 0, 1, 2, 3, 4, 5, 10, 15, 20, 25, and 30 min. The bacterial colonies were counted after incubation for 18 h.

A time kill kinetic assay was conducted to determine the time over which melectin acts on the bacteria. Figure 3 and Figure S2 show the time kill curves for melectin and melittin against *S. aureus* and *P. aeruginosa*. Melectin exerts its bactericidal effects very rapidly. Melectin killed more than 90% of *S. aureus* and 80% of *P. aeruginosa* in 5 min, eliminating nearly all bacteria within 20 min.

### Large unilamaller vesicle aggregation

**Figure 4:**
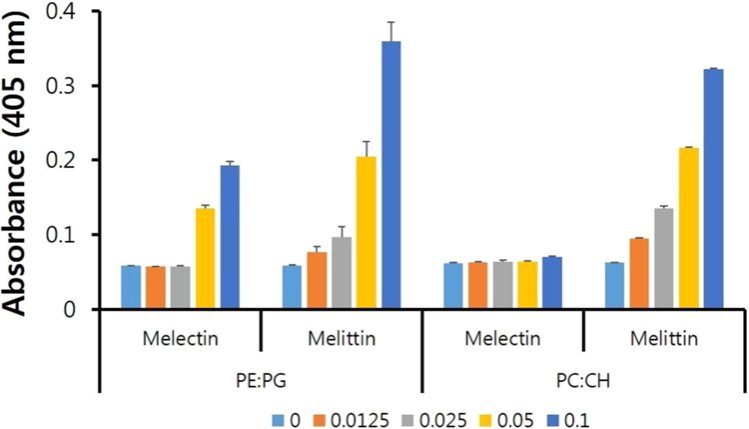
Liposome aggregation mediated by melectin. Aggregation of PE:PG (7:3, w/w) and PC:CH (10:1, w/w) with peptide/liposome ratios from 0.0125 to 0.1 measured as turbidity at 405 nm. PE,phosphatidylethanolamine; PG, phosphatidylglycerol; PC, phosphatidylcholine; CH, cholesterol.

Liposome turbidity was measured to evaluate peptide and liposome interactions according to the liposome interaction. Melectin induced aggregation of phosphatidylethanolamine (PE): phosphatidylglycerol (PG) (7:3, w/w), which is similar to a bacterial outer membrane. However, melectin did not induce liposome aggregation of phosphatidylcholine (PC): cholesterol (CH) (10:1, w/w), which is similar to erythrocytes. In PE:PG, the turbidity was increased when the ratio of peptide to liposome was 0.05. In contrast, the turbidity of PC:CH did not increase with increasing peptide/liposome ratios (Fig. 4).

### Activity in physiological salt concentration

Divalent or trivalent cations interfere with binding of the AMPs to the bacteria membrane. To use AMPs as therapeutic agents, their antimicrobial activity must be maintained at the physiological salt level. Thus, the antimicrobial activity of melectin was measured at physiological salt concentration. As a control, the MIC was 2 μM in 10 mM sodium phosphate buffer against *S. aureus* and *P. aeruginosa*. The antimicrobial activity of melectin against *S. aureus* ATCC 25923 was minimally affected by the presence of monovalent (Na^+^), divalent (Mg^2+^), and trivalent (Fe^3+^) cations. Melectin retained its antimicrobial activity of 2 μM at various salt concentrations. For *P. aeruginosa*, melectin showed slightly higher antimicrobial activity compared to *S. aureus*. Melectin retained its activity in the presence of monovalent and trivalent cations; however, the MIC value was 8 μM in the presence of a divalent cation (Mg^2+^) (Table 3).

**Table 3:** Minimum inhibitory concentration (MIC) of melectin at various salt concentrations.

MIC (μM)				
Peptides	Control^a^	NaCl^a^	MgCl_2_^a^	FeCl_3_^a^
*S. aureus* ATCC 25923				
Melectin	2	2	4	2
Melittin	2	2	4	2
*P. aeruginosa* ATCC 27853				
Melectin	2	4	8	2
Melittin	2	4	4	4

^a^The final concentrations of NaCl, MgCl_2_, and FeCl_3_ were 150 mM, 1 mM, and 4 μM, respectively, and the control was a 10 mM sodium phosphate buffer (pH 7.2)

### Mechanisms of peptide action

**Figure 5:**
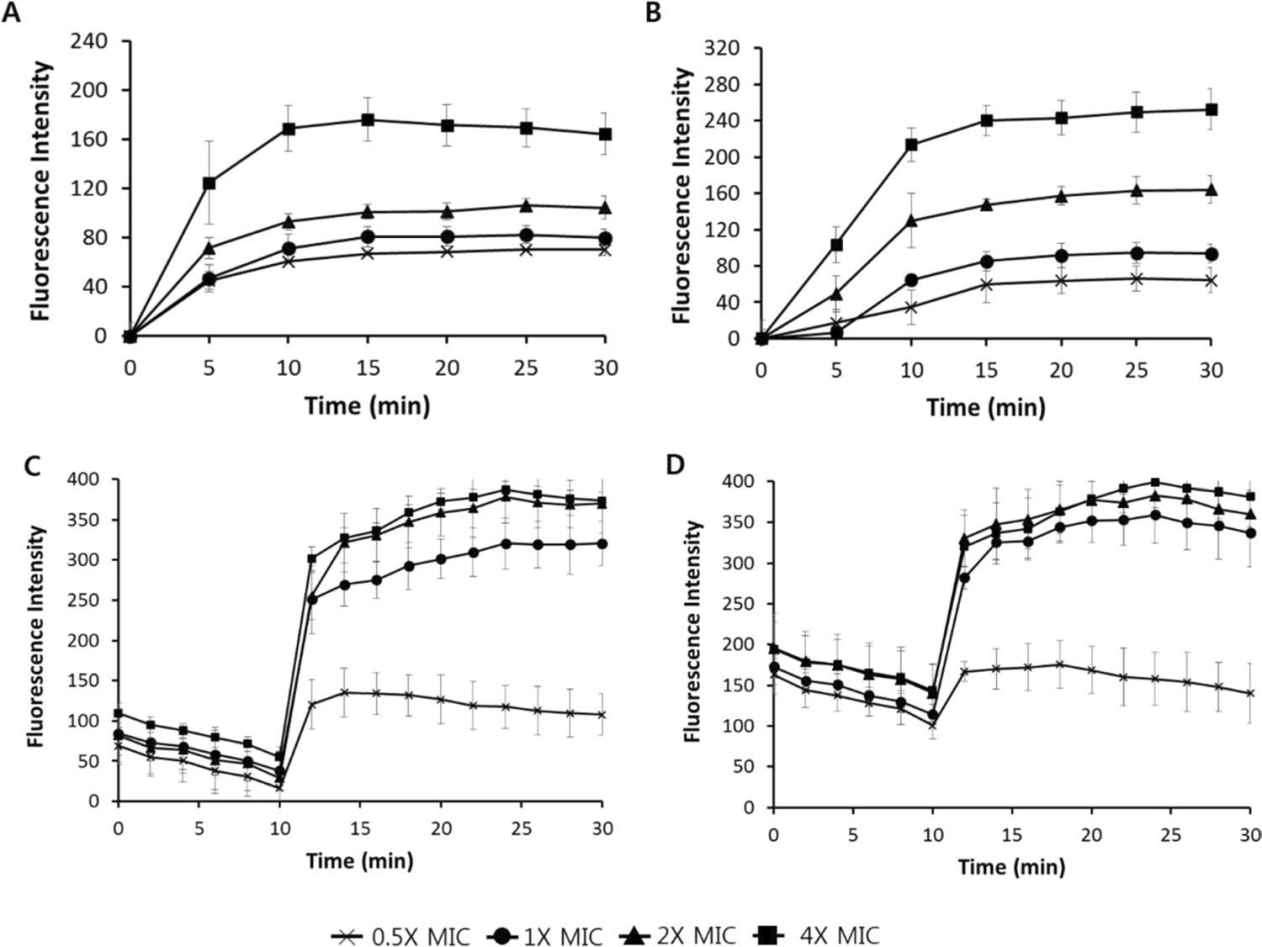
The effect of melectin on bacterial membrane. Outer membrane permeability determined as NPN uptake by (**A**) *S. aureus* ATCC 25923 and (**B**) *P. aeruginosa* ATCC 27853 in the presence of various concentrations of melectin. Cytoplasmic membrane depolarization of (**C**) *S. aureus* ATCC 25923 and (**D**) *P. aeruginosa* ATCC 27853 were measured using diSC_3_-5.

**Figure 6:**
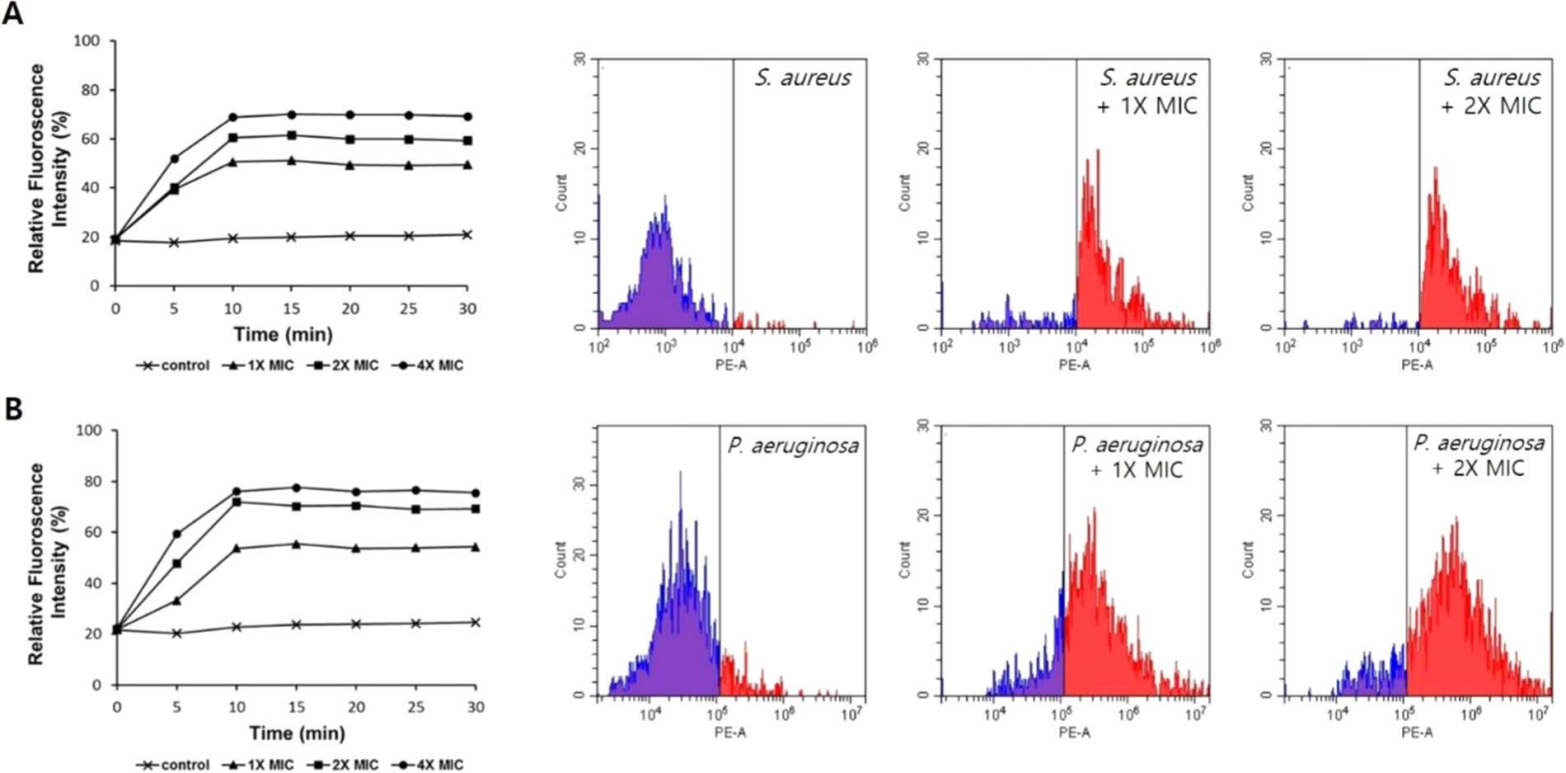
PI uptake assay and flow cytometric analyses of increases in PI fluorescence intensity after treatment with melectin. (**A**) *S. aureus* ATCC 25923, (**B**) *P. aeruginosa* ATCC 27853.

The outer membrane of bacteria plays a crucial role in protecting organisms. *n*-Phenyl-1-naphtylamine (NPN) uptake was measured to determine the ability of the antimicrobial peptide to permeabilize the outer membrane. When the outer membrane is damaged, NPN binds to the membrane and fluorescence is increased. As shown in Fig. 5A,B, melectin induced permeabilization of the *S. aureus* and *P. aeruginosa* membrane in a dose-dependent manner. Next, the membrane potential probe 3,3′-dipropylthiadicarbocyanine iodide (diSC_3_-5) was used to measure bacterial cytoplasmic membrane depolarization caused by melectin. diSC_3_-5 concentrates in the cytoplasmic membrane; when membrane is disturbed by the peptide, the cytoplasmic membrane electrical potential dissipates, after which diSC_3_-5 is released into the medium, resulting in a fluorescence increase. Melectin depolarized the bacterial cytoplasmic membrane (Fig. 5C,D). To confirm the mechanism of melectin, a propidium iodide (PI) uptake assay and flow cytometry were performed using PI, which fluoresces upon binding to nucleic acids. When bacteria are treated with a peptide, the peptide disrupts the bacterial membrane, causing PI to enter the membrane and increase fluorescence. Melectin induced an increase in PI fluorescence in a dose-dependent manner (Fig. 6). By flow cytometry, treatment with 1× and 2× MIC melectin resulted in percentages of PI staining of 82.4% and 92.7% against *S. aureus*. For *P. aeruginosa* with 1× and 2× MIC melectin, 79.6% and 85.8% of bacteria were stained with PI. Thus, melectin destroys the bacteria outer and cytoplasmic membranes.

### Melectin increases bacterial membrane permeability

**Figure 7:**
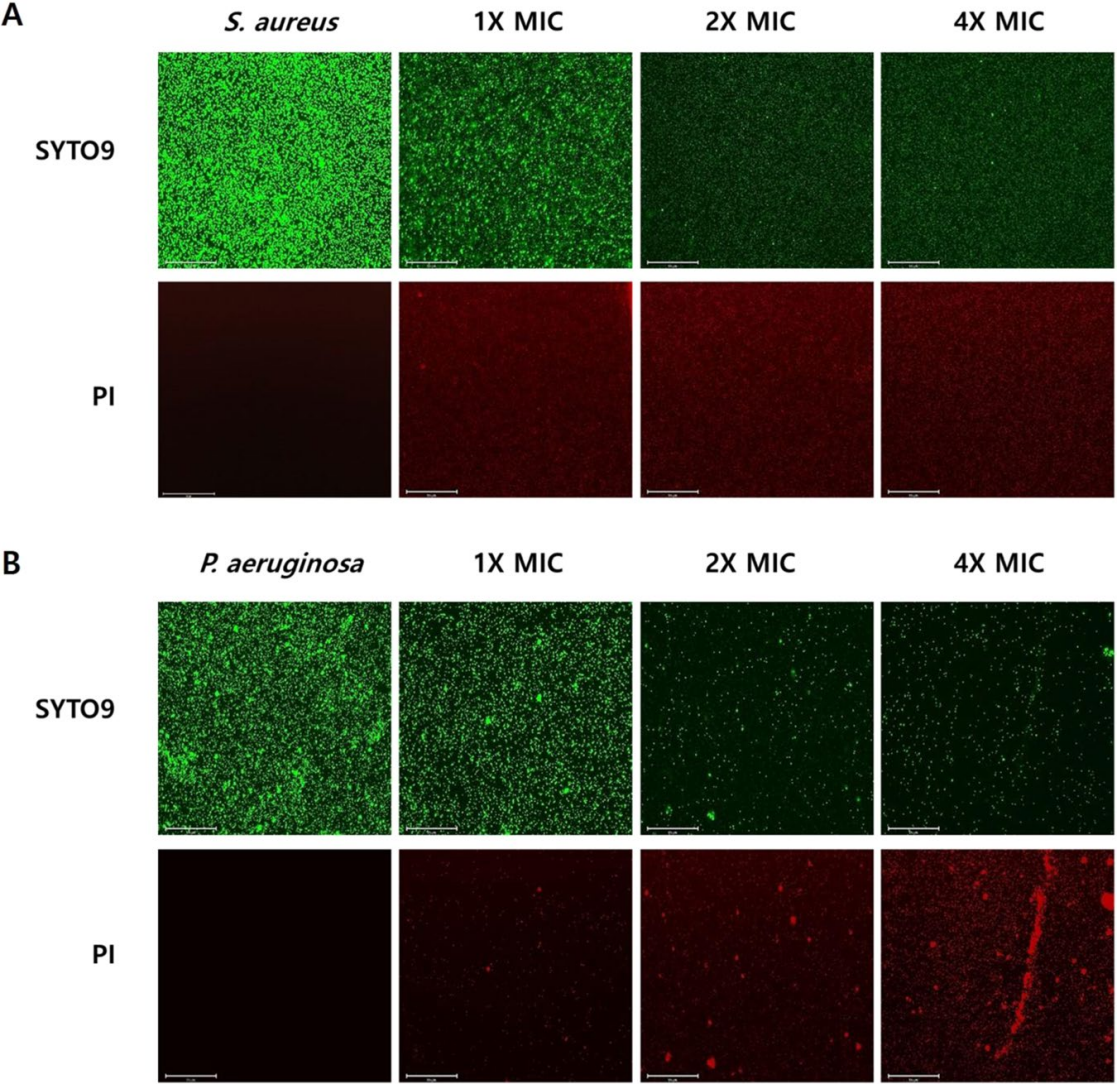
SYTO9/PI staining analyzed with microscopy. (**A**) *Staphylococcus aureus* ATCC 25923, (**B**) *P. aeruginosa* ATCC 27853 were treated with melectin and stained with SYTO9 and PI. Live bacteria appeared as green and dead bacteria appeared as red.

Membrane-permeable SYTO9 stains bacteria as green. In contrast, propidium iodide (PI) enters only damaged, nonliving cells. *Staphylococcus aureus* and *P. aeruginosa* were incubated with melectin, and SYTO9- and PI-stained bacteria were observed by microscopy. *Staphylococcus aureus* and *P. aeruginosa* appeared only as green, indicating that all bacteria were alive. However, bacteria treated with melectin showed red fluorescence, indicating membrane damage (Fig. 7). The degree of membrane permeabilization was increased as the melectin concentration was increased. Furthermore, the results indicated that the antimicrobial effect of melectin occurred through membrane permeabilization.

### Structure of melectin in the presence of LTA and LPS

**Figure 8:**
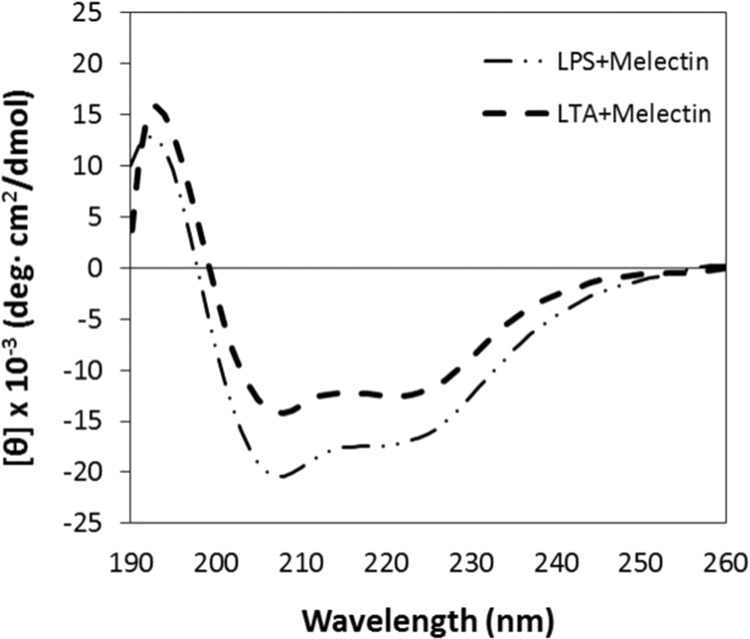
Interaction of melectin with LTA and LPS. Melectin was incubated with a 0.1% LTA aqueous suspension of *S. aureus* and a 0.1% LPS aqueous suspension of *P. aeruginosa*. The final concentration of the peptide was 40 μM.

LTA is major constituent of the cell wall of gram-positive bacteria, and LPS is found in the outer membrane of gram-negative bacteria. Circular dichroism (CD) spectroscopy was conducted to determine whether the peptide binds the negatively charged LTA or LPS. The degree of helicity of the peptide depends on their interactions and binding in the presence of LTA or LPS. Melectin with LTA and LPS showed negative bands at 208 and 222 nm, suggesting that the peptide binds to LTA and LPS and has an α-helical structure (Fig. 8).

### Inhibition of expression of proinflammatory cytokine

**Figure 9:**
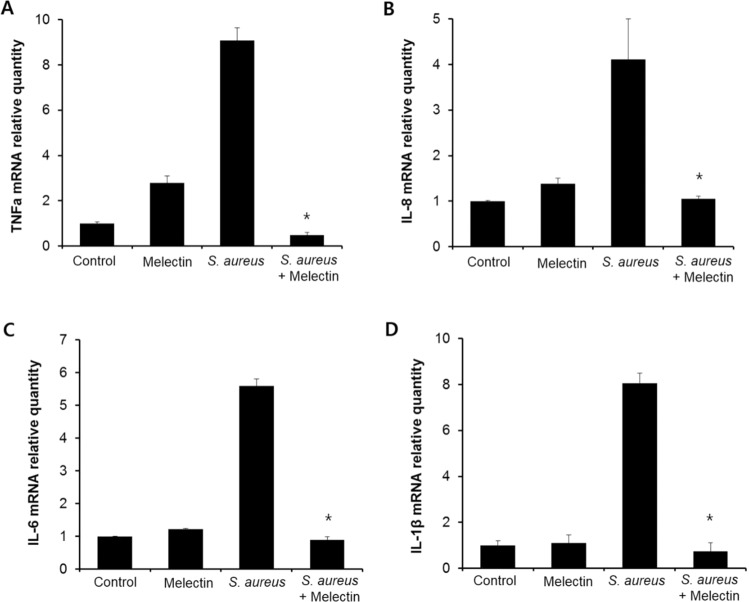
Effect of melectin on the release of TNFα, IL-8, IL-6, and IL-1β. Human fibroblasts were stimulated with *S. aureus* ATCC 25923 in the presence of 5 µM melectin for 24 h. Secreted TNFα, IL-8, IL-6, and IL-1β were measured through qRT-PCR. Three independent experiments were conducted, with each experiment being performed in triplicate. Means with a difference at p < 0.05 were considered significant.

**Figure 10:**
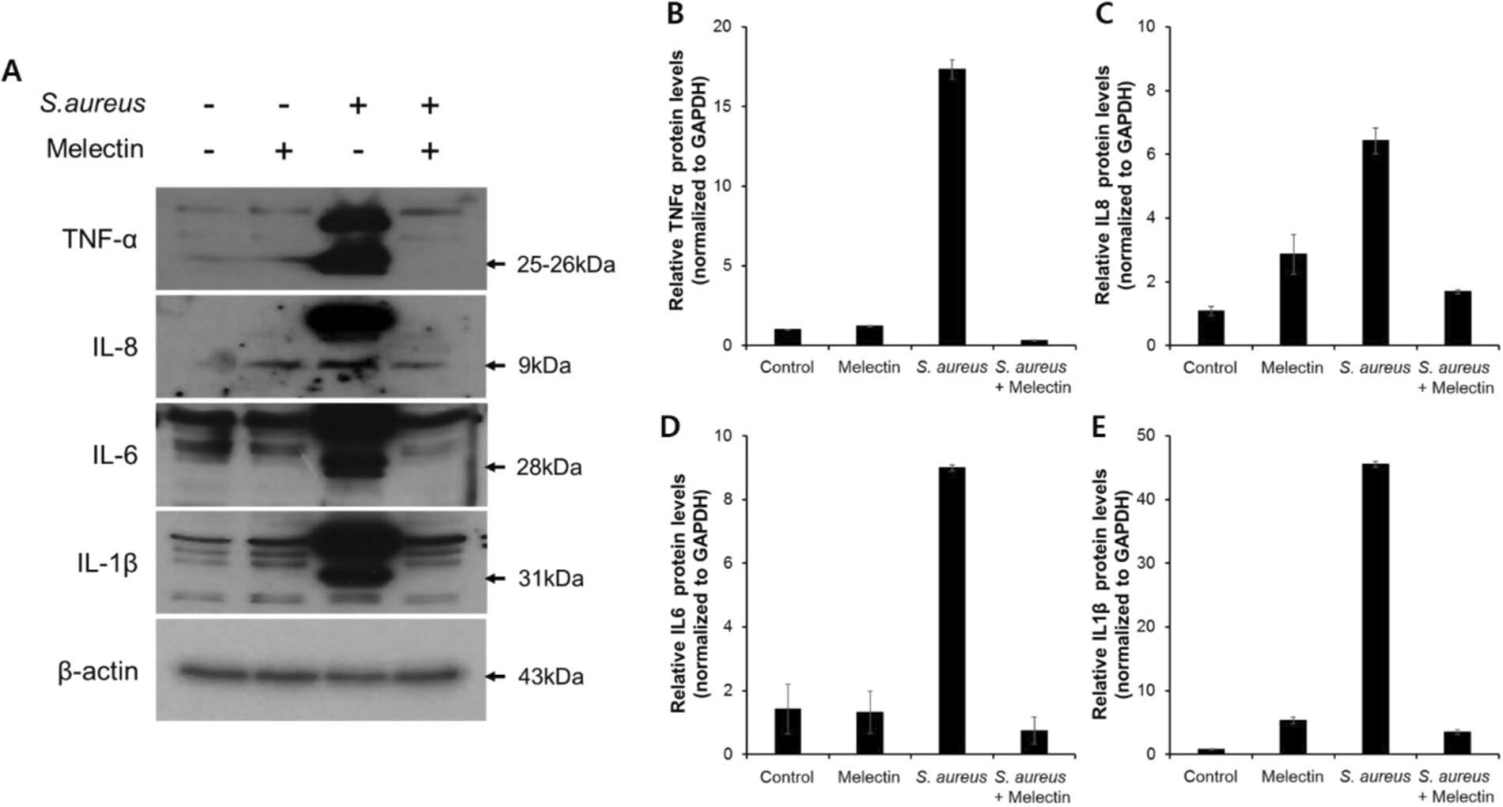
Western blot analysis of the effect melectin on levels of cytokine. (**A**) Human fibroblast were treated with *S. aureus* and melectin for 24 h. The cell lysates were analyzed for pro-inflammatory cytokine levels using western blotting analysis. The results were constructed as a bar graph (**B**), (**C**), (**D**), and (**E**).

To assess the effect of melectin on the release of TNFα, IL-8, IL-6, and IL-1β from *S. aureus*-stimulated human fibroblasts, the cells were treated with *S. aureus* in the presence or absence of melectin. *Staphylococcus aureus* induces mRNA and protein expression of pro-inflammatory cytokines such as TNFα, IL-8, IL-6, and IL-1β. When *S. aureus* was incubated with fibroblasts, the protein level of cytokines increased in a time-dependent manner (Fig. S3). Melectin significantly inhibited the increase in pro-inflammatory cytokines that were upregulated by *S. aureus* infection (Fig. 9 and Fig. 10). By contrast, melectin had no effect on basal TNFα, IL-8, IL-6, and IL-1β expression in uninfected fibroblasts. These results indicated that melectin inhibits the increase in inflammatory cytokines induced in fibroblast by infection.

## Discussion

Antimicrobial peptide melectin, consisting of 18 amino acid residues from solitary bee venom, has antimicrobial activity and has an α-helical structure^18,19^. Thus, we aimed to maintain the antimicrobial activity in physiological salt concentrations and induce anti-inflammatory activity.

The main function of bee venom is to protect against attacks from predators. Bee venom therapy using bee stings induces anti-inflammatory activity^20^. The components of bee venom have been extensively studied for their biological, toxicological, and pharmacological actions. The venom peptides mastoparan and melittin exhibit antimicrobial activity towards a broad range of bacteria^21,22^. Melittin is a representative bee venom peptide with 26 amino acids and is known to have excellent antimicrobial activity but high cytotoxicity in mammalian cells^23^. Melectin, which consists of 18 amino acids, has a shorter amino acid sequence than melittin but shows similar antibacterial activity. Furthermore, melectin has lower cytotoxicity and no hemolytic activity compared to melittin. Additionally, bactericidal kinetic data indicate that melectin approaches the bacterial membrane to kill the bacteria in a rapid manner.

A previous study showed that melectin has an α-helical structure in an environment mimicking the bacterial membrane, but a lower fraction of α-helical content was observed in an environment mimicking the eukaryotic membrane^19^. This is consistent with the results of the liposome aggregation experiments in the present study. The lipid bilayer of the bacterial membrane consists of a negatively charged phospholipid containing phosphatidylglycerol, cardiolipin, and phosphatidylserine. In contrast, mammalian cell membranes are mainly composed of zwitterionic phospholipids, including cholesterol, phosphatidylethanolamine, sphingomyelin, and phosphatidylcholine. The interaction between AMPs and mammalian cells, particularly erythrocytes, is unstable, and AMPs do not interrupt the mammalian membrane. Melectin induced aggregation of the PE:PG liposome but not the PC:CH liposome. Melectin is not cytotoxic towards mammalian cells and exhibits selectivity toxicity towards pathogens.

The antimicrobial activity of peptides is compromised at high salt concentrations^24^. Cationic peptides bind to a negatively charged bacterial membrane via electrostatic interactions. Cations such as Na^+^, Mg^2+^, and Fe^3+^ prevent the electrostatic interaction between the peptide and bacterial membrane, decreasing antimicrobial activity^25^. Thus, maintaining peptide activity in salt-containing environments is necessary condition for using peptides as therapeutic agents. We investigated the antimicrobial activity of melectin at physiological salt concentration. The results showed that melectin retained its antimicrobial activity in the presence of monovalent (Na^+^) and trivalent cations (Fe^3+^) and showed partial activity in the presence of divalent ions (Mg^2+^). Divalent cations may affect antimicrobial activity and appear to be strong peptide inhibitors^26^^,^^27^. Mg^2+^ interferes with the electrostatic interaction and competes for membrane binding, reducing AMP activity. The degree of influence on antimicrobial activity depends on the ion concentration. At high concentrations, divalent cations increased membrane rigidity through electrostatic interactions with the negatively charged phospholipid^28^. Some studies reported that AMPs with a low net charge were more affected by divalent cations than those with a high net charge^29^.

The first step in bacteriostasis is binding of the cationic peptide to the negatively charged constituents of the bacterial outer membrane. Next, the bacteria are destroyed by pore formation, destabilization of the membrane equilibrium, or penetration into the bacteria, causing depolarization and leakage of essential metabolites^30,31^. Studies of the association of AMPs with LTA or LPS are important for understanding the outer membrane permeability mechanism. To access the inner membrane or intracellular target, AMPs must bind to the gram-positive and gram-negative bacteria cell wall. Therefore, we measured the peptide interaction with LTA and LPS. To determine the mechanism of melectin, we conducted membrane permeability and depolarization experiments against gram-positive and gram-negative bacteria. Cationic melectin was shown to have high affinity for gram-positive (*S. aureus*) and gram-negative bacteria (*P. aeruginosa*). Melectin binds to the bacterial membrane and induces bacterial membrane permeabilization and depolarization as revealed in an NPN and Disc3-5 fluorescence assay. Furthermore, flow cytometry analysis indicated that melectin possessed antimicrobial activity through bacterial membrane destruction. Taken together, these results indicate that melectin binds to the bacterial outer membrane such as LTA or LPS, permeabilizes the bacterial membrane and depolarization, and kills the bacteria.

AMPs not only have antibacterial activity but also modulate bacterial-induced inflammatory responses^32^. Infection with bacteria increases the expression of pro-inflammatory cytokines including TNFα, IL-8, IL-6, and IL-1β, and cytokine overexpression causes inflammation and organ damage^33^. We found that pro-inflammatory cytokines were significantly increased when cells were treated with *S. aureus*. Melectin significantly inhibited the secretion of pro-inflammatory cytokine expression and their mRNA expression.

In this study, we investigated the antibacterial activity of melectin and its mechanism of action. Melectin and melittin used as a control in this study share a common peptide derived from bee venom. Melectin, which is composed of 18 amino acids, is shorter than the amino acid sequence of melittin (26 amino acids) but has low cytotoxicity while retaining its antimicrobial activity. Melectin exhibited excellent antimicrobial activity against gram positive and gram negative bacteria including drug-resistant bacteria without significantly cytotoxic towards mammalian cells. Additionally, melectin binds to LPS or LTA and kills bacteria through bacterial membrane permeabilization. Furthermore, melectin modulates the expression of pro-inflammatory cytokines induced by *S. aureus*. Overall, melectin is an antibacterial peptide with antibacterial and anti-inflammatory activities. This peptide has high potential for further research and application as a new drug.

## Materials and Method

### Materials

Rink amide 4-mehylbenzhydrylamine resin, N-hydroxybenzotriazole (HOBt), fluoren-9-ylmethoxycarbonyl (Fmoc) amino acids, and other reagents for peptide synthesis were purchased from Calbiochem-Novabiochem (La Jolla, CA, USA). Lipopolysaccharide (LPS) from *P. aeruginosa*, lipoteichoic acid (LTA) from *S. aureus*, 3,3′-dipropylthiadicarbocyanineiodide (DiSC3-5), N-phenyl-1-naphthylamine (NPN), and propidium iodide (PI) were obtained from Sigma-Aldrich (St. Louis, MO, USA).

### Microbial strains

The standard strain of the bacterira used in this study were obtained from the American Type Culture Collection (ATCC) and Korea Collection for Type Cultures (KCTC). Drug resistant strains (*S. aureus* 336, 254, and 660 and *P. aeruginosa* 3543, 1034, and 5018) were obtained from Chonnam National University Hospital (Gwangju, South Korea). The patients provided written informed consent for the use of their clinical isolates. *Escherichia coli* CCARM 1229 and *E. coli* CCARM 1238 were from the Culture Collection of Antibiotics Resistant Microbes at Seoul Women’s University, Korea.

### Peptide synthesis

The peptide was synthesized according to a solid-phase Fmoc method as reported previously^34^ on a Rink amide 4-methylbenzhydrylamine resin using a Liberty microwave peptide synthesizer (CEM Co., Charlotte, NC, USA). The crude peptide was purified by reverse-phase high-performance liquid chromatography on a Jupiter C18 column (4.6 × 250 mm, 300 Å, 5 μm). Peptides were separated using a gradient of 10–60% acetonitrile for 40 min at a flow rate of 1 mL/min. Elutes were monitored by measuring the absorbance at 280 min. The molecular mass of the peptide was confirmed using a matrix-assisted laser desorption/ionization time-of-flight mass spectrometer (Kratos Analytical, Inc., Chestnut Ridge, NY, USA)^35^.

### Antimicrobial Activity

Antimicrobial activity assay to determine the minimum inhibitory concentration (MIC) of melectin as described by the National Committee of Clinical and Laboratory Standard methods^36^. *Staphylococcus aureus* ATCC 25923, *Escherichia coli* ATCC 25922, *Pseudomonas aeruginosa* ATCC 27853, *Salmonella typhimurium* KCTC 1926, *Klebsiella pneumoniae* KCTC 2208, and antibiotic-resistant strains were incubated in culture medium at 37 °C. Aliquots of bacterial cell suspension (2 × 10^5^ CFU/mL) were added to a flat-bottomed 96 well plate. The peptide was serially diluted in 10 mM sodium phosphate buffer. The samples were incubated at 37 °C for 18 h in an incubator. The MIC of the peptide that inhibited bacterial growth was determined by measuring the optical density (OD) at 600 nm using a microplate reader. Each measurement was performed at least three times using two replicates. This study was exempt from ethical approval according to the Bioethics Law (Article 36 (2) of the Act to Article 33 of the Enforcement Rules).

### Cell Culture of HDF

Human Dermal Fibroblasts (HDF) were purchased from Gibco™ (Grand Island, NY). HDF were subcultured with Dulbecco’s modified Eagle’s medium (DMEM) supplemented with 10% fetal bovine serum and antibiotics at 37 °C in a humidified chamber under 5% CO_2_.

### Cytotoxicity Assay

The MTT assay was conducted to determine the cytotoxicity of the peptide as previously described^37^. The cells were seeded into a flat-bottomed 96-well plate at 2 × 10^4^ cells/well in DMEM and incubated overnight. The following day, the peptide was added to cells in concentrations ranging from 0 to 32 μM. After incubation, 0.5 mg/mL MTT was added to each well and incubated for 4 h. The supernatants were removed and 100 μL of DMSO was added to dissolve the formazan crystals. Cytotoxicity was measured using a microplate reader at 570 nm. The control was DMEM without peptide. Each measurement was performed at least three times using two replicates.

### Hemolytic activity

To confirm the hemolytic activities of the peptides, sheep red blood cells were washed with phosphate-buffered saline (PBS) and the supernatant was cleared by centrifugation at 1000 ×*g* for 5 min. The peptide was serially diluted at a concentration of up to 32 μM in PBS and placed in each well of a flat-bottomed 96-well plate. Next, 4% red blood cells were added to each well at a final volume of 200 μL. The plate was incubated for 1 h at 37 °C, and the reacted samples were centrifuged at 1000 ×*g* for 15 min. The supernatant was transferred to a new 96-well plate and detected by measuring the optical density (OD) at 414 nm (Molecular Devices, Sunnyvale, CA, USA). The percentage of hemolysis was calculated as follows: hemolysis (%) = (Abs_sample_ – Abs_PBS_)/(Abs_triton_ – Abs_PBS_) × 100. To determine the zero hemolysis and 100% hemolysis values, PBS and 0.1% Triton X-100, respectively, were added to the red blood cells.

### Preparation of large unilamellar vesicles (LUVs) and aggregation

Large unilamellar vesicles (LUVs) were prepared by the freeze-thaw method. The liposome mixture was prepared by dissolving in chloroform with PE: PG (7:3. w/w) and PC: CH (10:1, w/w), and the dissolved liposomes were dried using argon gas. Next, lyophilization was performed under high vacuum to completely remove chloroform residues and the dried lipid films were resuspended in PBS by vortexing for 30 min. LUVs were prepared using nine freeze-thaw cycles under liquid nitrogen and in a water bath. The suspensions were extruded through a 0.2-μm polycarbonate membrane. The lipid concentration was determined using a standard phosphate assay.

Liposome aggregation was confirmed by measuring turbidity. Melectin and melittin were prepared at 5, 10, 20, and 40 μM and added to 400 μM LUVs of PC:CH (10:1, w/w) to mimic the erythrocyte membrane or PE:PG (7:3. w/w) to mimic the bacterial outer membrane. Aggregation of the peptide and liposome increased the turbidity, the absorbance of which was measured at 405 nm.

### Stability assay

To determine if the presence of different salts affects peptide stability, *S. aureus* and *P. aeruginosa* were incubated overnight and diluted to 2 × 10^5^ CFU/mL in MHB. The peptide was serially diluted to concentrations of 0–62 μM in the presence of 150 mM NaCl, 1 mM MgCl_2_, and 4 μM FeCl_3_^38^. The following steps were performed as described for determining the MIC. Each measurement was performed at least three times using two replicates.

### Kinetics of bacterial killing

A final concentration of 2 × 10^5^ CFU/mL of *S. aureus* and *P. aeruginosa* was treated at 1X MIC concentration of peptide for various periods (1, 2, 3, 4, 5, 10, 15, 20, 25, and 30 min) at 37 °C. Aliquots of each sample were plated onto an MHB agar plate. The bacterial colonies were counted after 18 h of incubation. Each measurement was performed at least three times using two replicates.

### Evaluation of outer membrane permeability

Outer membrane permeability caused by peptide was determined by NPN uptake^39^. *Staphylococcus aureus* and *P. aeruginosa* were grown at 37 °C in MHB. The bacteria were washed three times and re-suspended to an optical density at 600 nm (OD_600_) of 0.2, using 5 mM HEPES buffer (pH 7.2). Next, the bacteria were placed in a black 96-well plate and NPN was added to the bacteria at a final concentration 10 μM. Additionally, the peptides at 0.5×, 1×, 2×, and 4× of the MIC were added to the plate and measured for 30 min at 5-min intervals. The excitation and emission wavelengths were 305 and 420 nm, respectively. Each measurement was performed at least three times using two replicates.

### Cytoplasmic membrane electrical potential measurement

The cytoplasmic membrane depolarization by peptide was determined using the membrane potential-sensitive fluorescent dye diSC_3_-5^40^. *Staphyloccocus aureus* and *P. aeruginosa* were incubated at 37 °C in MHB and washed with washing buffer (5 mM HEPES and 20 mM glucose). The bacteria were then resuspended to an optical density at 600 nm of 0.05 with 5 mM HEPES buffer containing 20 mM glucose, which included 0.1 M KCl, to equilibrate the cytoplasmic and external K + . Subsequently, the diSC_3_-5 dye was added to the bacteria at a final concentration of 1 μM and the mixture was incubated for 1 h to stabilize the fluorescence degree. The peptide at 0.5×, 1×, 2×, and 4× of the MIC was added to the mixture of bacteria and diSC_3_-5. The excitation and emission wavelengths were 622 and 670 nm, respectively. Each measurement was performed at least three times using two replicates.

### PI uptake assay

*Staphylococcus aureus* and *P. aeruginosa* were cultured in MHB and diluted to an OD_600_ value of 0.25 in 10 mM sodium phosphate buffer (pH 7.2) containing MHB medium. The bacteria were mixed with PI to a final concentration 20 μM in a black 96-well plate. After mixing, a 200-μL aliquot was treated at 1×, 2×, and 4× of the MICs of the peptide. The control was added to only 10 mM sodium phosphate buffer. PI fluorescence was monitored at excitation and emission wavelengths of 580 and 620 nm, respectively, over time. Each measurement was performed at least three times using two replicates.

### Flow cytometry

Integrity of the bacteria membranes by peptide was determined by flow cytometry. *S. aureus* and *P. aeruginosa* were cultured in MHB and washed three times with PBS, and diluted to an OD_600_ value of 0.5. The 1× and 2× MICs of melectin were added, and the mixture was incubated for 30 min at 37 °C. The bacteria were harvested by centrifugation and washed with PBS, and then incubated with a final concentration of 10 μg/mL PI for 30 min at 4 °C. Next, unstained PI was washed away with PBS. The data were recorded by flow cytometry (Beckman Coulter, Brea, CA, USA).

### Fluorescence microscopy

*Staphylococcus aureus* and *P. aeruginosa* were cultured and diluted to an OD_600_ value of 0.2 in MHB media. Bacteria were reacted with the peptide at 1×, 2×, and 4× MICs for 30 min at 37 °C. After incubation, the mixture was centrifuged at 7,000 × g for 10 min and the supernatant was discarded. The pellets were pipetted with 1 mL of PBS; 10 μM propidium iodide and 1 μM SYTO 9 were added and reacted for 15 min in the dark. Next, the cells are harvested by centrifugation and released with a small amount of PBS, and then 10 μL of cells were placed on slide glass and covered with a cover slip, and observed using a phase contrast microscope (EVOS^TM^ FL Auto 2 Imaging System, Thermo Fisher Scientific, Waltham, MA, USA).

### LTA and LPS binding assay

Circular dichroism spectrum analysis was conducted to analyze the secondary structure of the peptide in different solutions mimicking the bacterial membrane environment. The solutions were prepared with lipopolysaccharide (components of outer membrane of Gram-negative bacteria), and lipoteichoic acid (components of outer membrane of Gram-positive bacteria). The peptide was prepared at a concentration of 40 μM. CD spectra were measured between 190 and 250 nm using a JASCO 810 spectropolarimeter (Jasco, Tokyo, Japan) with a 1.0-mm quartz cuvette.

### RNA isolation and quantitative real-time PCR (qRT-PCR)

Human dermal fibroblasts (5 × 10^5^ cell/mL) were seeded into a 6-well plate and incubated overnight. Next, the fibroblasts were incubated with *S. aureus* (1 × 10^8^ CFU/well) for 24 h in the presence or absence of 5 µM melectin. Total RNA was isolated using a TRIzol RNA extraction kit (Life Technologies, Carlsbad, CA, USA) according to the manufacturer’s protocol, and then reverse-transcribed into cDNA using a Topscript^TM^ cDNA synthesis kit (Enzynomics, Daejeon, Korea). Target gene mRNA expression was analyzed with the 7500 RT-PCR system using SYBR Green (Applied Biosystems, Foster City, CA, USA). To quantify the mRNA, transcript levels were normalized to the level of *GAPDH* mRNA.

### Western Blotting Analysis

After lysis of fibroblasts in lysis buffer and centrifugation at 20000 × *g* for 15 min at 4 °C, the protein concentration in the supernatants were determined using the Bradford assay. The proteins were separated using SDS-polyacrylamide gel electrophoresis and transferred onto polyvinylidene fluoride (PVDF) membranes. The membranes were blocked with 5% skim milk in Tris-buffered saline containing Tween-20 (TBST) for 1 h. Then, the membranes were incubated with primary antibodies overnight at 4 °C: anti-TNFα antibody (1:500 dilution, Invitrogen, Carlsbad, CA, USA), anti-IL-8 antibody (1:500 dilution, Invitrogen, Carlsbad, CA, USA), anti-IL-6 antibody (1:500 dilution, Thermo Scientific, Waltham, MA, USA), anti-IL-1β (1:500 dilution, Waltham, MA, USA), anti-β-actin antibody (1:1000 dilution, Santa Cruz, Dallas, Texas, USA). After washing with TBST, the membranes were incubated with the secondary antibodies for 2 h. After washing with TBST, the reaction was detected using ECL solution (Bio RAD, Hercules, CA, USA).

### Statistical analyses

All experiments were performed in triplicate in at least three independent experiments. Error bars represent the mean ± SD. Differences with *P* < 0.05 were considered statistically significant.

## Supplementary information

Supplementary information.

## Data Availability

The authors declare no restrictions on the availability of materials or information.
