# Supplementary material for: Bee venom-derived antimicrobial peptide melectin has broad-spectrum potency, cell selectivity, and salt-resistant properties
Source: Sci Rep. 2020 Jun 23;10:10145. doi: 10.1038/s41598-020-66995-7 (PMC7311438; doi:10.1038/s41598-020-66995-7)
Supplement: Supplementary file 1 — Supplementary information. [file 41598_2020_66995_MOESM1_ESM.docx]

**Bee venom-derived antimicrobial peptide melectin has broad‑spectrum potency, cell selectivity, and salt-resistant properties**

**Su Jin Ko^1^, Eunji Park^1^, Alina Asandei^2^, Jee-Young Choi^3^, Seung-Chul Lee^3^,** **Chang Ho Seo^4^,** **Tudor Luchian^2,*^, Yoonkyung Park^1,5,*^**

^1^ *Department of Biomedical Science, Chosun University, Gwangju 61452, South Korea*

*^2^ Department of Physics, Alexandru I. Cuza University, Iasi, Romania*

*^3^ Departments of Dermatology, Chonnam National University Medical School, Gwnagju, Korea*

*^4^ Department of Bioinformatics, Kongju National University, Kongju, Republic of Korea, 32588*

^5^*Research Center for Proteineous Materials, Chosun University, Gwangju 61452, South Korea*

^*^Corresponding authors.

E-mail addresses: [y_k_park@chosun.ac.kr](mailto:y_k_park@chosun.ac.kr) (Y. Park), [luchian@uaic.ro](mailto:luchian@uaic.ro) (T. Luchian)

**
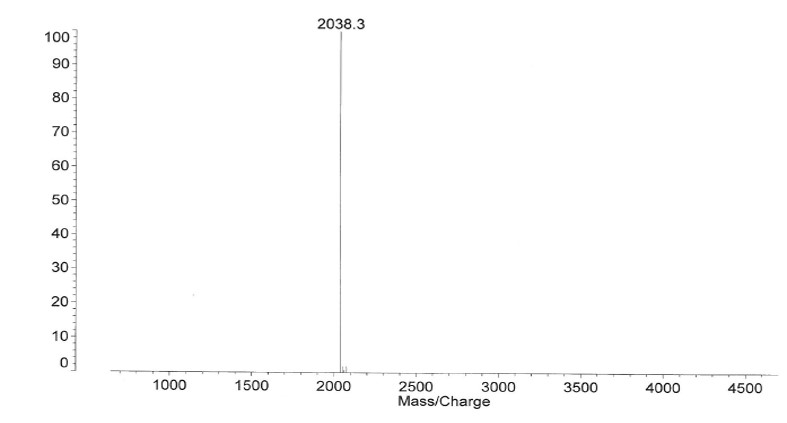
Figure S1. MALDI-TOF mass spectrum of melectin.**

**Table S1.** Minimum inhibitory concentration (MIC) of antibiotics against resistant bacterial strains`

| **Minimum Inhibitory Concentration (μM)** | | | | |
| --- | --- | --- | --- | --- |
|  | Oxacillin | Gentamicin | Ampicillin | Ciprofloxacin |
| *S. aureus* MRSA 254 | >256 | >256 | >256 | >256 |
| *S. aureus* MRSA 366 | >256 | 4 | >256 | 4 |
| *S. aureus* MRSA 660 | >256 | >256 | >256 | >256 |
| *P. aeruginosa* 1034 | >256 | 128 | >256 | >256 |
| *P. aeruginosa* 3543 | >256 | 128 | >256 | >256 |
| *P. aeruginosa* 5018 | >256 | 128 | >256 | >256 |
| *E. coli CCARM* 1229 | >256 | 64 | >256 | 16 |
| *E. coli* CCARM 1238 | 256 | 16 | >256 | 4 |

**
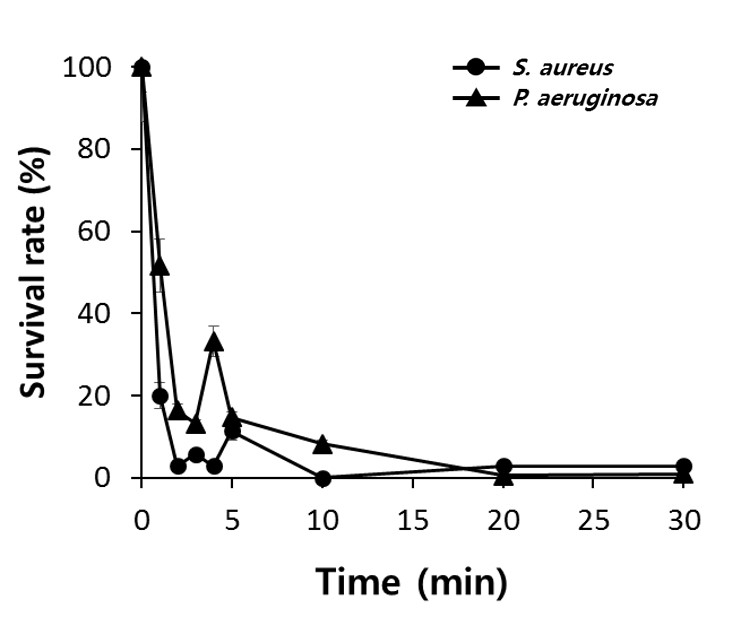
**

**Figure S2. Time-kill kinetic curves of melittin against microorganisms.** *S. aureus* ATCC 25923 and *P. aeruginosa* ATCC 27853 were exposed to melittin for 0, 1, 2, 3, 4, 5, 10, 15, 20, 25, and 30 min.

**
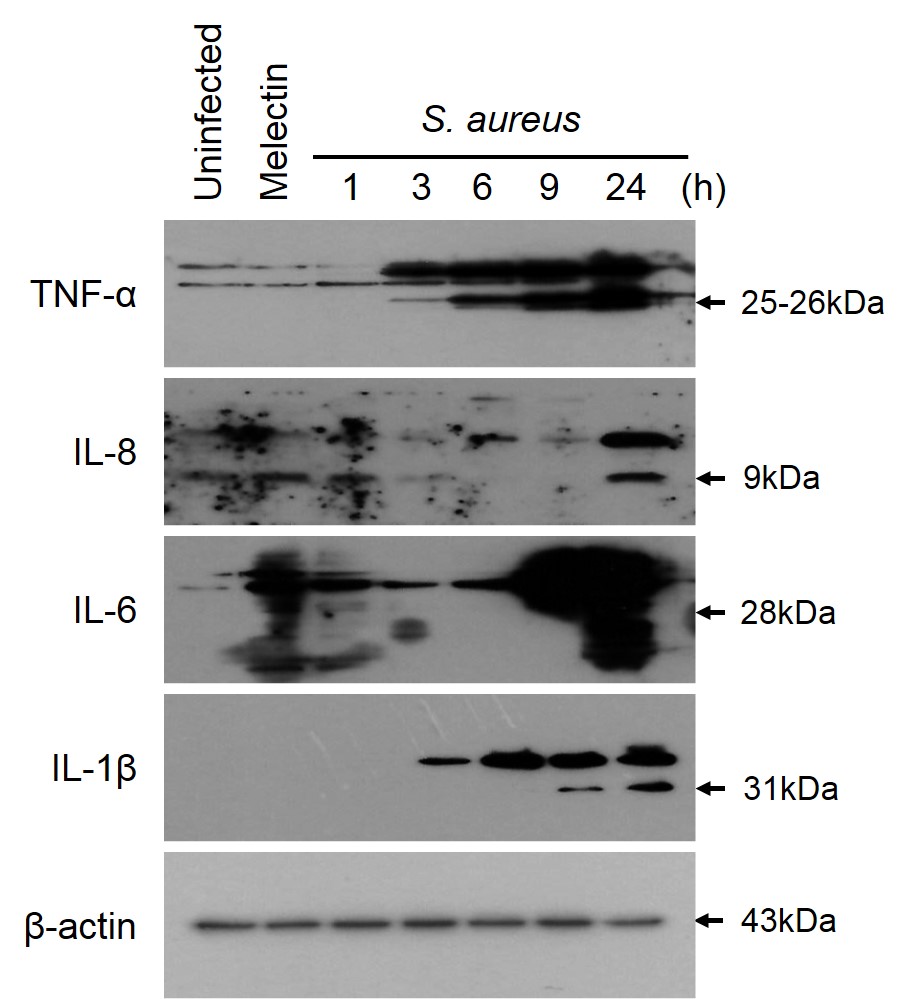
**

**Figure S3. Human fibroblasts were stimulated with *S. aureus* for 1, 3, 6, 9, and 24 h.** The cell lysates were analyzed using western blotting.


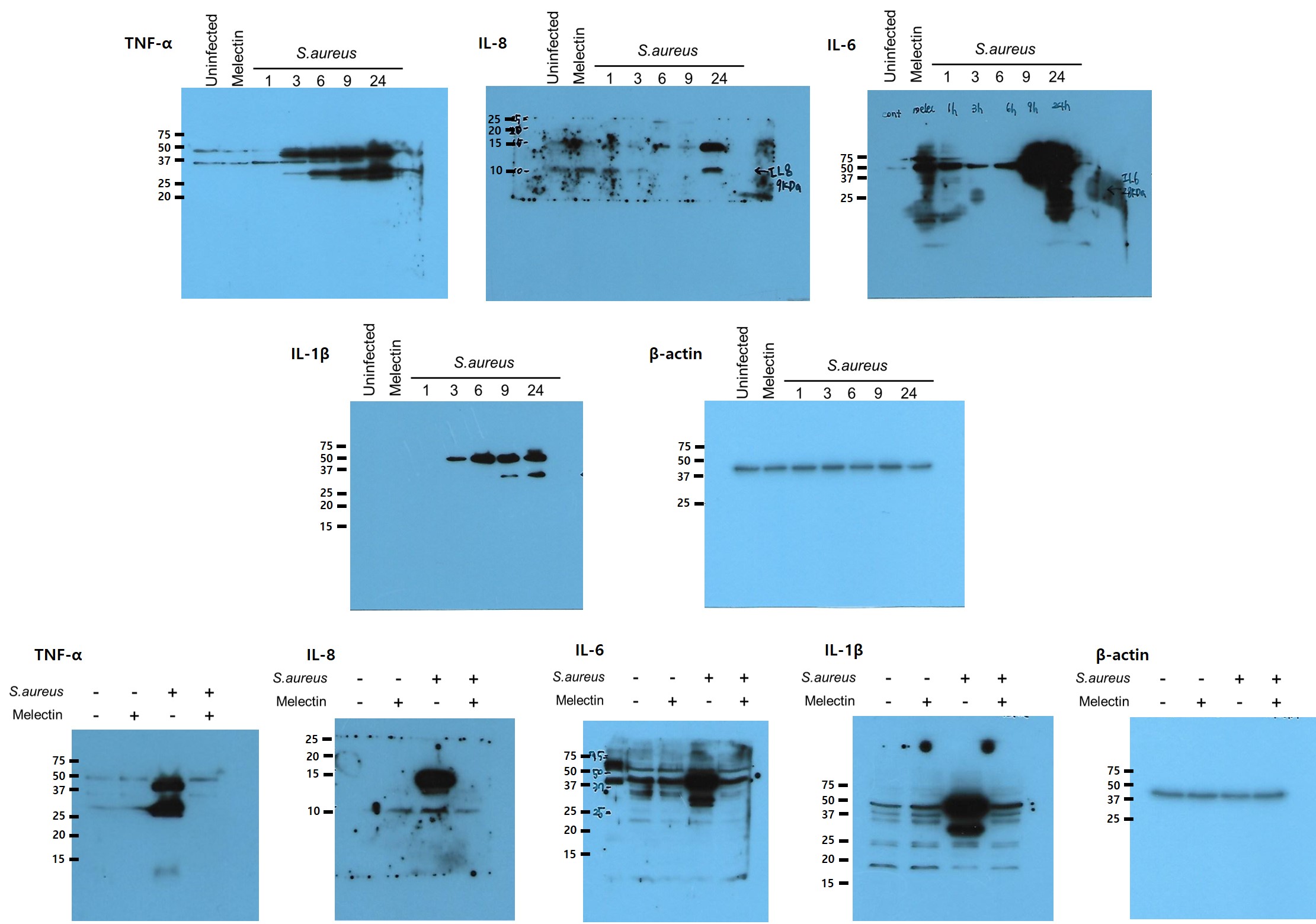


**Figure S4. Uncropped western blotting images.**
